# A Review of the Literature on Extrarenal Retroperitoneal Angiomyolipoma

**DOI:** 10.1155/2016/6347136

**Published:** 2016-02-17

**Authors:** Anthony Kodzo-Grey Venyo

**Affiliations:** North Manchester General Hospital Department of Urology, Delaunay's Road, Manchester, UK

## Abstract

*Background*. Extrarenal retroperitoneal angiomyolipomas are rare.* Aim*. To review the literature.* Results*. Angiomyolipomas, previously classified as hamartomas, are now classified as benign tumours. Thirty cases of primary retroperitoneal angiomyolipomas have been reported. Diagnosis of the disease upon is based radiological and pathological findings of triphasic features of (a) fat and (b) blood vessels and myoid tissue. Immunohistochemistry tends to be positive for HMB45, MART1, HHF35, calponin, NKI-C3, and CD117. The lesion is common in women. Treatment options have included the following: (a) radical surgical excision of the lesion with renal sparing surgery or radical nephrectomy in cases where malignant tumours could not be excluded and (b) selective embolization of the lesion alone or prior to surgical excision. One case of retroperitoneal angiomyolipoma was reported in a patient 15 years after undergoing radical nephrectomy for angiomyolipoma of kidney and two cases of distant metastases of angiomyolipoma have been reported following radical resection of the tumour.* Conclusions*. With the report of two cases of metastases ensuing surgical resection of the primary lesions there is need for academic pathologists to debate and review angiomyolipomas to decide whether to reclassify angiomyolipomas as slow-growing malignant tumours or whether the reported cases of metastases were de novo tumours or metastatic lesions.

## 1. Introduction

Extrarenal retroperitoneal angiomyolipomas (ERAMLs) also referred to as retroperitoneal angiomyolipomas (RAMLs) by some authors are rare lesions which may mimic other retroperitoneal tumours and thus pose a diagnostic problem to the clinician. There are on the whole three tissue components that constitute angiomyolipomas (AMLs) including mature adipose tissue, thick-walled blood vessels, and smooth muscle cells [1, 2]. AMLs were previously classified as hamartomas; however, AMLs are now regarded as belonging to the family of perivascular epithelioid cell tumours (PEComas) [1, 2]. Rare cases of ERAMLs have been reported. It has been documented that ultrasound scan and CT scan can diagnose correctly AMLs of the kidney in 86% of cases [3]. Nevertheless, ERAMLs are more difficult to diagnose on imaging in view of the fact that they tend to lack fat densities [4]. The ensuing review of the literature on extrarenal retroperitoneal angiomyolipoma (ERAMLs) is divided into two parts: (A) overview and (B) miscellaneous narrations and discussions from reported cases of extrarenal retroperitoneal angiomyolipoma and an article related to treatment of angiomyolipoma.

## 2. Methods

Various internet data bases were searched for the literature on extrarenal retroperitoneal angiomyolipoma (retroperitoneal angiomyolipoma) including Google, Google Scholar, PubMed, and Educus. The search words that were used included extrarenal retroperitoneal angiomyolipoma, retroperitoneal angiomyolipoma, and angiomyolipoma. Information obtained from 69 references mainly related to retroperitoneal angiomyolipoma and one on treatment of angiomyolipoma using mTor inhibitor rapamycin was used to write the literature review.

## 3. Literature Review

### 3.1. Overview (A)

#### 3.1.1. General Comments about Angiomyolipomas (AMLs)

The following has been stated [33]:

- AMLs tend to be benign neoplasms which comprise thick-walled vessels, smooth muscles, and fat (adipose) tissue with spindle and epithelioid cells but the amount of each of the aforementioned components is variable in different AMLs [33].
- AMLs may occur in the kidney and at times AMLs may be found in extrarenal sites. In 2012 Minja et al. [18] summarized the extrarenal sites of AMLs they had encountered in their literature review as follows: uterus (7 cases) [34], hard palate (1 case) [35], head (2 cases) [36], abdominal wall (1 case) [37], penis (1 case) [38], fallopian tube (1 case) [39], liver (18 cases) [40], nasal cavity (1 case) [41], vagina (2 cases) [42, 43], spermatic cord (1 case) [44], and colon (1 case) [45], and the retroperitoneum (16 cases) [18]. A total of 52 cases of extrarenal angiomyolipomas had been reported according to Minja et al. [18] at the time of publication of their paper in 2012.
- The occurrence of AML could be sporadic; nevertheless, AML has been associated with epiloia (tuberous sclerosis) and also with TSC/PKD1 contiguous gene syndrome [33].

#### 3.1.2. Epidemiology

- It has been stated that AML tends to be diagnosed in adults and that AMLs of the kidney constitute less than one percent of kidney tumours [33].
- The ages of the patients who have been reported to have been diagnosed with extrarenal retroperitoneal angiomyolipoma (ERAML), also called retroperitoneal angiomyolipoma (RAML) by other authors, have ranged between 22 years and 80 years (see Table 1).
- The occurrence of AML in the retroperitoneum is rare and to the knowledge of the author 30 cases have been reported in the literature and the retroperitoneum constitutes the second most common site of extrarenal angiomyolipoma [18].
- Extrarenal retroperitoneal angiomyolipoma (ERAML), also called retroperitoneal angiomyolipoma (RAML) by other authors, has been reported more commonly in females in comparison with males. Out of the 30 cases of extrarenal retroperitoneal angiomyolipomas (ERAMLs) reported the sex of the patients was available in 25 cases of which 24 (84%) were reported in women and 4 (16%) were reported in men (see Table 1).

#### 3.1.3. Pathophysiology

- Stone et al. [46] stated that angiomyolipoma (AML) is a member of the epithelioid cell (PEC) AML which includes pulmonary lymphangioleiomyomatosis; clear cell sugar tumours of the pancreas, lung, and uterus; and also rhabdomyomas of the heart [33].
- It has also been stated that angiomyolipomas (AMLs) are neoplasms and not hamartomas and that a number of cases of angiomyolipomas (AMLs) have loss of heterozygosity of TSC2 gene [33].

#### 3.1.4. Molecular Genetic Studies

- Cheng et al. [47] iterated that smooth muscle cells and adipose tissue tend to be monoclonal but they may arise independently. Cheng et al. [47] reported that they had undertaken a study in which smooth muscle, adipose tissue, blood vessels, and adjacent normal tissue of the kidney were separately microdissected from formalin-fixed, paraffin-embedded, processed tissues of angiomyolipomas of 18 women. They studied the clonal origin of each component of angiomyolipoma by means of X chromosome inactivation analysis by using the methylation pattern of exon 1 of the human androgen receptor gene on chromosome Xq11-12. Cheng et al. [47] reported that they found nonrandom inactivation of X chromosomes in six out of the 15 informative tumours; the smooth muscle and adipose tissue did show differing patterns of nonrandom inactivation of X chromosomes in 5 angiomyolipomas and the same pattern of nonrandom inactivation of one; samples taken from blood vessels did show random inactivation of X chromosomes in all of the informative cases. Cheng et al. [47] concluded that their data had illustrated that the adipose tissue and smooth muscle cells of angiomyolipoma of the kidney are both monoclonal but they may arise independently; the coexistence of tumour subclones with morphologic heterogeneity could lead to the formation of a clinically detected tumour.

#### 3.1.5. Clinical Characteristics

- Angiomyolipomas (AMLs) occur in the kidney but sporadic cases occur in other sites above with the liver being the commonest site where the tumour tends to have dominant epithelioid smooth muscle component [33].
- It has been stated that angiomyolipomas (AMLs) may be found contemporaneously in association with renal cell carcinoma in cases of nonepiloia (nontuberous sclerosis) patients, especially clear cell carcinoma of the kidney which tends to stain negatively on immunohistochemistry studies [33, 48].
- Angiomyolipomas (AMLs) tend to be benign; nevertheless, they may be associated with haemorrhage, invasion of nearby organs, or involvement of organs that are not contiguous [33].
- Angiomyolipomas (AMLs) tend to have characteristic features on radiological imaging which helps in the establishment of the diagnosis [33].

#### 3.1.6. Tuberous Sclerosis

- Tuberous sclerosis is an autosomal dominant neurocutaneous disorder which occurs in 1 out of 6 thousand to 11 thousand individuals and it is characterized by the developments of hamartomas/tumours in the brain (subependymal giant cell tumour), retina, skin (angiofibromas of skin), bone, lung (lymphangioleiomyomas, multifocal micronodular pneumocyte hyperplasia), and renal (angiomyolipoma in 40% to 80%, cysts, and renal cell carcinoma; nevertheless, some epithelioid tumours may be unclassified) as well as mental retardation and infantile/childhood convulsions [33].
- Tuberous sclerosis tends to be caused by alterations of TSC1 gene (9q34, which encodes hamartin) as well as TSC2 gene (16p13.3 which encodes tuberin that interacts with hamartin) [33].
- It has been stated that, in TSC/PKD1 contiguous gene syndrome, both the left and the right kidneys tend to be enlarged and cystic and they tend to be associated with classic angiomyosarcomas and rare intraglomerular microlesions [33, 49].

#### 3.1.7. Presentation

Extrarenal retroperitoneal angiomyolipoma (ERAML) may manifest in a variety of ways including the following (see Table 1):

- as an incidental finding, following radiological imaging investigation for various symptoms,
- pain in the abdomen, loin, or back,
- history of gain in weight,
- fullness in epigastrium and tiredness,
- feeling of fullness in the abdomen or abdominal distension and pain,
- pain and bleeding,
- haematuria,
- vomiting or constipation,
- abdominal mass and weight loss,
- there may or may not be a history or clinical features of tuberous sclerosis.

#### 3.1.8. Laboratory Investigations

*(1) Hematological Investigations*. Full blood count and coagulation screen are basic investigations that are undertaken in the assessment of a patient who has extrarenal retroperitoneal angiomyolipoma (ERAML); however, there is nothing specific in the tests that would be diagnostic of angiomyolipoma (AML).

*(2) Serum Biochemistry Investigations*. Serum urea and electrolytes/renal function tests, liver function tests, bone profile, and serum glucose are basic tests that are undertaken in the assessment of a patient with angiomyolipoma (AML) but none of the test results are specific for the diagnosis of extrarenal retroperitoneal angiomyolipoma (ERAML) which has also been referred to by other authors as retroperitoneal angiomyolipoma (RAML).

*(3) Urinalysis, Urine Microscopy, and Culture*. These tests are basic tests undertaken in the assessment of the patient but they are not investigations that would diagnose angiomyolipoma (AML).

#### 3.1.9. Radiological Investigations

*(1) Ultrasound Scan*

- Ultrasound scan of the abdomen and pelvis can be undertaken which would tend to show the mass in the retroperitoneum; it would also reveal the size and extent of the tumour and show whether or not the tumour mass has displaced any nearby organ.
- If there is hydronephrosis due to obstruction of the ureter by the angiomyolipoma (AML) tumour mass, the ultrasound scan would demonstrate it.
- In cases of hydronephrosis plus or minus impaired renal function, ultrasound guided insertion of percutaneous nephrostomy can be undertaken to help improve renal function or avoid deterioration in renal function.
- Ultrasound of abdomen and pelvis may also demonstrate on rare occasions a metastatic lesion in the liver. Gupta et al. [4] stated that ultrasound scan is useful for the detection of angiomyolipoma (AML).
- Antegrade ureteric stenting can be undertaken following insertion of nephrostomy to help the surgeon identify the ureter during surgical excision/resection of the angiomyolipoma (AML) that has encased the ureter to avoid ureteric injury during the surgical procedure.
- Ultrasound guided biopsy of the angiomyolipoma (AML) can also be undertaken.

*(2) Computed Tomography (CT) Scan*. Minja et al. [18] stated the following:

- CT scan and CT angiography (CTA) are the commonest used imaging techniques in the investigation of angiomyolipomas (AMLs) [18].
- Wang et al. [50] undertook an analysis of the radiological characteristics of CT scans of the abdomen in cases of extrarenal retroperitoneal angiomyolipomas (ERAMLs) and observed that extrarenal retroperitoneal angiomyolipomas (ERAMLs) typically tend to show aneurysmal dilatation of the intramural vessels, intramural linear vascularity, bridging veins beak sign, hematomas, and discrete intrarenal/extrarenal fatty tumours, but none of these are pathogenic.
- CT scans of the brain have been recommended in relation to patients who have angiomyolipomas (AMLs) of the kidney, considering the fact that 30% to 40% of such patients may also have CT brain scan features of epiloia (tuberous sclerosis) and likewise 80% of such patients would end up developing angiomyolipoma (AML) of the kidney [51–53]. The CT scan of the brain in such patients typically tends to reveal characteristic periventricular subependymal nodules with calcifications [15].
- CT-guided biopsy of the retroperitoneal mass can be undertaken for pathological examination

*(3) Magnetic Resonance Imaging (MRI) Scan*

- Minja et al. [18] stated that MRI scan could be utilized in addition to CT and that MRI scan is particularly useful for the delineation of the anatomical relationship between extrarenal retroperitoneal angiomyolipomas (ERAMLs), the kidney, as well as its vasculature, particularly in cases of perinephric and retroperitoneal angiomyolipomas.
- MRI-guided biopsy of the retroperitoneal mass can be undertaken to obtain specimen for histological examination.

*(4) PET/CT Scan*

- In extrarenal retroperitoneal angiomyolipoma (ERAML), PET/CT scan would demonstrate a hypermetabolic extrarenal mass in an extrarenal location which on further investigation by means of pathological examination of the specimens obtained from the excised specimen or radiological imaging guidance biopsies would confirm the diagnosis of angiomyolipoma (AML) [22].
- PET/CT scan done as part of an investigation of a different condition could lead to the incidental finding of a retroperitoneal mass [22].

#### 3.1.10. Macroscopic Features

It has been stated that gross examination of angiomyolipomas (AMLs) tends to show red areas in the vascular component, gray-white areas in the smooth muscle component, and yellow areas in the adipose component of the tumour which mimic clear cell carcinoma [33].

It has also been iterated that gross inspection of angiomyolipoma (AML) may reveal evidence of invasion of local lymph nodes and renal vein by the tumour even though the tumour is benign, and invasion of the capsule of the tumour may be observed in 25% of cases [33, 54].

Angiomyolipomas (AMLs) on gross examination tend to be found in unilateral positions and they also tend to be unifocal [33].

In angiomyolipomas (AMLs), gross inspection tends to reveal multiple tumours in one-third of the cases or bilateral in 15% of the tumours which would be suggestive of epiloia (tuberous sclerosis) [33].

On rare occasions of angiomyolipoma (AML) there may be evidence of gross or microscopic cysts [33, 55].

#### 3.1.11. Microscopic Features

- Microscopic examinations of angiomyolipomas (AMLs) classically trend to reveal triphasic features which include myoid spindle cells, islands of mature adipose tissue (fat), and dysmorphic blood vessels that are thick-walled and which do not have elastic lamina [33].
- The smooth muscle component of angiomyolipomas (AMLs) on microscopic examination tend to appear to have originated from walls of vessels and they may appear to be hypercellular, atypical, pleomorphic, or epithelioid [33].
- On microscopic examination, angiomyolipoma (AML) may mimic a high grade sarcoma if it metastasizes [33].
- With regard to epithelioid variant of angiomyolipoma (AML), microscopic examination of the lesion tends to show pure or predominant population of large, epithelioid cells that have clear or eosinophilic cytoplasm, large hyperchromatic bizarre-looking nucleus, and possibly multinucleation which include an intimate relationship with vessel wall [33].
- Microscopic examination of angiomyolipomas (AMLs) show common areas of haemorrhage and necrosis [33].
- Microscopic examination of angiomyolipoma (AML) may reveal adipose tissue which could be scanty or dominant and this may mimic well-differentiated liposarcoma [33].
- Microscopic examination may show small mesenchymal nodules that are less than 2 cm which are precursors of angiomyolipoma (AML) [56].
- Microscopic examination of angiomyolipoma (AML) may show epithelial cysts [55, 57] and prominent sclerosis [58].

#### 3.1.12. Cytology Features

- Cytological examination of specimens of angiomyolipoma (AML) tends to reveal oval to spindled cells and cohesive stromal fragments, adipose tissue, and branching blood vessels within a haemorrhagic background [33].
- Cytological examination of angiomyolipomas (AMLs) also tend to show mitotic figures [59].

#### 3.1.13. Immunohistochemistry of Angiomyolipomas (AMLs)

*(1) Positive Staining*. In angiomyolipoma (AML) immunohistochemical studies of adipose tissue, myoid, and epithelioid cells tend to show positive staining with regard to various markers as follows:

- They are HMB45 (100%) [60], MART1/Melan-A, muscle specific actin (HHF35, 100%), calponin (100%), and NKI-C3 (70–100%) [33].
- CD117 tends to be positive [61], desmin tends to be positive in 20% of cases [62], HMB50 in 100% of cases, microphthalmia transcription factor in 50% of cases [63], and progesterone receptor in 28% of cases and these tend to occur in women who are aged less than 50 years and associated with tuberous sclerosis, smooth muscle actin, and tyrosinase in 20% to 50% of cases, and in vimentin [33].
- Immunohistochemistry tends to show evidence of lymphatic differentiation podoplanin and D2-40 [64] and S100 (fat component) [33].
- HMB45 and Melan-A tend to be positive in descending order of percentage positivity in fat, followed by in smooth muscle and then followed by blood vessels [65].

*(2) Negative Staining*. Immunohistochemical staining of angiomyolipoma (AML) tends to be negative for Keratin and renin [33].

#### 3.1.14. Molecular Characteristics

*5q-Changes*. Kattar et al. [66] stated that angiomyolipoma had previously been stated to be a hamartomatous polyclonal proliferation. Nevertheless, recent molecular analysis studies had indicated that angiomyolipomas (AMLs) may be clonal neoplasms rather than polyclonal proliferations. Kattar et al. [66] investigated the chromosomal imbalances in angiomyolipoma (AML) by comparative genomic hybridization. They extracted DNA from paraffin-embedded and frozen tissues of 12 angiomyolipomas (10 usual variants and 2 epithelioid variants). The 10 angiomyolipomas (AMLs) of the usual variant included bilateral tumours from one tuberous sclerosis patient. Fluorescence ratio distributions from tumour hybridizations were compared with those from control hybridizations to detect changes in DNA copy number with sensitivity and specificity. Kattar et al. [66] identified 20 chromosomal imbalances in 7 sporadic angiomyolipomas (AMLs) which included both tumours of the epithelioid variant. The remaining 6 tumours including the two angiomyolipomas (AMLs) from a tuberous sclerosis patient lacked chromosomal imbalances. Seventy percent of the imbalances were partial or whole chromosomal deletions involving disparate genomic regions, some of which had earlier been reported to be associated with tumours of adipose tissue and smooth muscle tumours. Four angiomyolipomas (AMLs) of the usual variant had shown 5q deletions with a common region of deletion spanning 5q33 to q34. In two of the tumours, deletion on 5q was the sole abnormality. One epithelioid angiomyolipoma showed 5q gain encompassing the same region in addition to other alterations. Kattar et al. [66] concluded that (I) chromosomal imbalances are common in angiomyolipomas (AMLs) of the kidney; (II) presence of clonal genomic alterations would additionally be supportive of the neoplastic pathogenesis of these tumours; (III) the 5q33-q34 region may contain a tumour suppressor gene significant in the histogenesis of some kidney angiomyolipomas.

#### 3.1.15. Monoclonal Proliferation of an Uncommitted Cell

Paradis et al. [67] stated that angiomyolipomas (AMLs) of kidney had been considered as hamartomas but little data had been available concerning their pathogenesis. It had not been known for sure if angiomyolipoma (AML) is a congenital malformation or a neoplastic process. In order to answer the aforementioned question, Paradis et al. [67] assessed the clonality of sporadic angiomyolipoma (AML) using molecular analysis. Seven women with a mean age of 59 years with angiomyolipoma (AML) of the kidney were included in the study. DNA of the tumour and the normal adjacent kidney was extracted from paraffin-embedded tissue. Paradis et al. [67] studied the DNA methylation pattern at a polymorphic site on the HUMARA gene by polymerase chain reaction (PCR) amplification after methylation-sensitive enzyme digestion. This procedure does enable the differentiation between polyclonal and monoclonal lesions according to the X chromosome inactivation pattern. Five of the 7 women included in the study were informative for the HUMARA gene. The mean size of the AMLs was 53 mm (this had ranged from 18 mm to 110 mm). In one of the cases, a tumour thrombus was found in the inferior vena cava. Clonal analysis had shown that all the angiomyolipomas (AMLs) and the tumour thrombus studied were monoclonal lesions consistent with neoplastic disorders. Paradis et al. [67] concluded that the results strongly support the postulate that angiomyolipomas (AMLs) arise from the clonal proliferation of an uncommitted cell, which will further evolve towards different cell types.

#### 3.1.16. Electron Microscopic Feature

Electron microscopic examination of angiomyolipoma (AML) tends to show premelanosomes [33].

#### 3.1.17. Differential Diagnoses

Some of the lesions that may mimic angiomyolipoma (AML) include the following:

- oncocytoma in which microscopic examination of the lesion tends to show prominent oncocytes, no evidence of prominent adipose component, and immunohistochemistry study which is negative for melanocyte markers [33],
- leiomyoma in which microscopic examination of the lesion tends to show no evidence of prominent vascular or adipose component and the immunohistochemistry study of the lesion tends to be negative for melanocytic markers [33],
- leiomyosarcoma in which microscopic examination of the lesion tends to show infiltrative lesion, evidence of prominent atypia, and quite commonly no prominent vascular or adipose component and immunohistochemistry studies of the lesion tend to be negative for melanocytic markers [33],
- melanoma in which microscopic examination of the lesion tends to show marked atypia, no evidence of prominent adipose, or vascular component in the lesion [33],
- pleomorphic rhabdomyosarcoma in which microscopic examination of the lesion tends to show smooth muscle component which is often an infiltrative tumour that is markedly atypical, no evidence of prominent adipose, or vascular component and immunohistochemical staining of the tumour tends to show negative staining for melanocytic markers [33],
- renal cell carcinoma in which microscopic examination of the lesion tends to show marked atypia and infiltrative margins and no evidence of being triphasic, and immunohistochemistry study of the lesion tends to be negative for melanocytic markers [33].

#### 3.1.18. Treatment 

Minja et al. [18] stated the following:

- The primary treatments for extrarenal retroperitoneal angiomyolipomas (ERAMLs) have commonly involved surgery (surgical excisions but less often embolization of the tumour have been undertaken).
- Surgical excision is indicated in cases of symptomatic, complex appearing, radiologically enlarging, or large extrarenal retroperitoneal angiomyolipomas (ERAMLs), which tend to be associated with a higher potential to bleed spontaneously.
- With regard to the patients who present in emergency situations symptomatically as a result of spontaneous retroperitoneal bleeding, selective arterial angiography and embolization have been undertaken effectively to control bleeding from haemorrhagic lesions in patients who are hemodynamically unstable which tend to result in involution of the tumour and which tend to allow for subsequent elective surgical excision or clinical observation (see [12, 14, 16]).
- Surgical excision has always been recommended in order to allow for histological examination to differentiate suspected extrarenal retroperitoneal angiomyolipomas (ERAMLs) from the differential diagnoses of lesions involving the retroperitoneum. The establishment of definitive diagnosis by means of pathological examination of surgically resected specimens of extrarenal retroperitoneal angiomyolipomas (ERAMLs) would dictate the length and type of appropriate follow-up considering that extrarenal retroperitoneal angiomyolipomas (ERAMLs), unlike malignant retroperitoneal sarcomas or renal/adrenal carcinomas which may require long-term surveillance.

#### 3.1.19. Follow-Up of Patients

Angiomyolipomas (AMLs) and extrarenal angiomyolipomas (ERAMLs) used to be defined as hamartomas but these lesions have subsequently been classified as benign tumours. Considering the fact that angiomyolipomas have been documented to have metastasized to lymph node and to the liver it is the opinion of the author that angiomyolipomas should be regarded as tumours that often exhibit benign biological behaviour but a subset of such tumours would metastasize; therefore, perhaps angiomyolipomas should be regarded as slow-growing malignant tumours with the potential to metastasize. In view of the fact that angiomyolipomas could metastasize it would be recommended that whether or not patients with extrarenal retroperitoneal angiomyolipomas (ERAMLs) have been treated by selective angiography and superselective embolization or surgical excision the patients should be followed up over a long period of time by means of appropriate radiological imaging that would minimize extensive cumulative radiation and perhaps a 5-year follow-up may be sufficient.

#### 3.1.20. Outcome

With regard to the outcome of ERAMLs following treatment, Minja et al. [18] stated the following:

- Out of the cases they had reviewed, 56% of the patients had been followed up over a period of time which had ranged between 2 months and 60 months after they had undergone surgical excision of their tumours.
- Outside the context of epiloia (tuberous sclerosis), they had encountered only one reported case of tumour recurrence up to the time of publication of their paper which occurred pursuant to a radical en bloc nephrectomy with the development of distant metastasis to the liver and bone 12 months after the surgical operation [4]. The rest of the patients had remained asymptomatic and free of disease at their last follow-up, and there had not been any documentation of recurrence of disease following a renal sparing nephrectomy or embolization.
- Considering the fact that the only recurrence they found in their review of the literature had occurred 12 months after an en bloc radical nephrectomy, they were of the opinion that extrarenal retroperitoneal angiomyolipomas (ERAMLs) should be followed closely with CT scans during the first year after surgical excision of the lesions, with continued yearly follow-up for 5 years or the duration of follow-up should be dictated by the symptoms of the patient.

### 3.2. Miscellaneous Narrations and Discussions from Some Reported Cases of Retroperitoneal Angiomyolipoma (B)

Friis and Hjortrup [5] in 1982 reported a 22-year-old woman who presented with pain and weight gain. She had intravenous urography which revealed an extrarenal retroperitoneal mass. For which she underwent exploratory laparotomy with radical nephrectomy including excision of the mass in the peripancreatic space and histological examination of the specimen which weighed 11 kilograms revealed features consistent with angiomyolipoma (AML). She was asymptomatic at her 30 months' follow-up. They stated that angiomyolipoma (AML) is a benign tumour and its treatment should always be surgical in view of the fact that it is difficult to establish the differential diagnosis preoperatively. Nevertheless, angiomyolipoma (AML) has a tendency toward recurrence if the entire tumour is not removed [5].

Randazoo et al. [6] in 1987 reported a 64-year-old woman with epiloia (tuberous sclerosis) who presented with abdominal pain due to spontaneous rupture of a 6 cubic centimetres retroperitoneal angiomyolipoma situated in the right perinephric space which was diagnosed by means of intravenous urography and CT scan of abdomen. She underwent right renal sparing excision of the mass. Pathological examination of the tumour mass showed features consistent with angiomyolipoma (AML). The patient was well at her 2 months' follow-up. Randazoo et al. [6] stated that their case was the first case of extrarenal angiomyolipoma associated with tuberous sclerosis.

Ditonno et al. [7] in 1992 reported two cases of extrarenal angiomyolipoma. One of the two cases involved a 37-year-old man who presented with abdominal pain. He had intravenous urography, CT scan of abdomen, and angiography which revealed 5 cm bleeding right extrarenal mass in the perinephric space. He underwent radical nephrectomy. Pathological examination finding of the specimen was consistent with a diagnosis of angiomyolipoma (AML). His follow-up data was not available.

Peh et al. [8] in 1994 reported a 32-year-old woman who presented with weight loss and abdominal mass. She had ultrasound scan and CT scan of the abdomen which revealed a mass in her left perinephric space. She underwent left radical nephrectomy which included the mass lesion which weighed 3.7 kilograms. Pathological examination of the specimen revealed features that were adjudged to be consistent with a diagnosis of angiomyolipoma (AML). She was asymptomatic at her 8-month follow-up.

Angulo et al. [9] in 1994 reported a 53-year-old female who presented with abdominal and left loin pain. She has ultrasound scan, CT scan, and angiography which showed a mass in the left perinephric space 336 cubic centimetres. She underwent left radical nephrectomy and pathological examination finding of the specimen was consistent with a diagnosis of angiomyolipoma (AML). There was no follow-up data available on the patient.

Liwnicz et al. [11] in 1994 reported a 39-year-old woman who presented with abdominal pain. She had a CT scan which showed a mass in her right perinephric space. She underwent a right radical nephrectomy with removal of a 1.1 kilogram mass. Histological examination of the specimen revealed features that had been adjudged to be consistent with angiomyolipoma (AML). Pathological examination of the tumour revealed tumour fat on the whole which was inconspicuous and manifested largely as hibernoma-like microvesicular lipid. The tumour cells also did on Immunohistological staining showed that the tumour cells were positively stained for HMB-45 and S-100 protein. The cells on electron microscopy studies showed occasional cytoplasmic striated granules which were indistinguishable from stage II premelanosomes. Nevertheless, electron microscopy and immunohistochemistry studies of the tumour also did confirm the presence of a substantial myogenous component of the tumour which established the diagnosis of AML. She was asymptomatic at her 18 months' follow-up.

Law et al. [12] reported two cases as follows.

**Case 1 .**

Law et al. [12] in 1994 reported a 59-year-old woman who was found incidentally to have a mass in her left perinephric space when she had CT scan and MRI scan of the abdomen and pelvis. She underwent a left radical nephrectomy with excision of a 22.5 cubic centimetres mass. Pathological examination of the specimen revealed features that were adjudged to be consistent with the diagnosis of angiomyolipoma (AML). Her follow-up outcome data was not available.

**Case 2 .**

Law et al. [12] in 1994 reported a 56-year-old woman who presented with abdominal pain. She had a number of investigations including intravenous urography, ultrasound scan of abdomen and pelvis, and CT scan of abdomen and as well as fine needle aspiration of a mass which was found in her left perinephric space. She underwent left radical nephrectomy and histological examination of the excised 11 cm mass revealed features that were consistent with a diagnosis of angiomyolipoma (AML). She was asymptomatic at her 8-month follow-up.

Mogi et al. [13] in 1998 reported a 41-year-old woman who 2 years earlier undergone surgery for type IIc early gastric cancer and who presented with back pain and fullness of her abdomen. She had CT scan and MRI scan of her abdomen and pelvis which revealed a massive fat tumour mass that had extended from the hepatic hilus to the retroperitoneum (in her right perinephric space as well as her perihepatic space). She underwent renal sparing excision of a 648 cubic centimetres mass located in her right perinephric and perihepatic space and the pathological examination of the specimen revealed features consistent with angiomyolipoma (AML) which did not involve the kidney. Her follow-up outcome data was not available.

Murphy et al. [14] in 2000 reported a 51-year-old woman who presented with abdominal pain due to bleeding from her angiomyolipoma (AML). She had a CT scan of her abdomen and angiography which revealed a bleeding from a mass in her left perinephric space. She underwent selective angiography and superselective embolization of the mass which had radiological imaging characteristics of angiomyolipoma (AML). She was asymptomatic at her 12-month follow-up.

Tsutsumi et al. [15] in 2001 reported a 60-year-old man who presented with fullness in the epigastrium and tiredness. A smooth, round soft painless mass was palpable on his abdominal examination. He had CT scan of her abdomen and pelvis which revealed a 22 cm × 22 cm × 10 cm lobulated fatty mass around the right kidney and a small fatty mass in the left kidney. The tumour was well delineated from the surrounding organs. She had abdominal angiography which demonstrated that the mass which had scattered aneurysmal lesions was fed by the right renal, adrenal, and lumbar arteries. He had a CT scan of the brain which did show multiple small calcified subependymal nodules which had extended from the lateral margins into both ventricles that was suggestive of tuberous sclerosis. He underwent en bloc resection of the mass together with the right kidney via a thoracoabdominal incision with radical nephrectomy with excision of 3.5 kilogram mass. The tumour was noted to be well encapsulated and was easily dissected from the surrounding tissues as well as it was noted that the regional lymph nodes had not been involved. Pathological examination of the specimen revealed features consistent with angiomyolipoma (AML) in that microscopic examination of the tumour revealed that the tumour comprised of mature fat cells which contained smooth muscle and thick-walled blood vessels, all of which characteristically typify angiomyolipoma (AML). He was alive and asymptomatic at her 60-month follow-up. Tsutsumi et al. [15] stated that the diagnostic dilemma of the tumour could be solved by performing renal arteriography and CT scan of the brain and in their case the two studies had been helpful in that the renal arteriogram had revealed the aneurysmal dilatation of the intratumoural vessels, which typifies angiomyolipoma (AML), and the CT scan of the brain did show periventricular subependymal nodules with calcification which had be indicative of tuberous sclerosis.

Tseng et al. [16] in 2004 reported a 35-year-old woman who presented with symptom of fullness in her abdomen. She had ultrasound scan and CT scan of her abdomen and pelvis as well as angiography which revealed a mass in her right perinephric space (retroperitoneum). She underwent arterial embolization and renal sparing excision of the lesion which weighed 2.8 kilograms. Pathological examination of the specimen revealed features consistent with angiomyolipoma (AML). Her follow-up outcome data was not available.

Obara et al. [17] in 2005 reported a 31-year-old man who presented with painless visible haematuria. He had a CT scan of abdomen which revealed a large perinephric mass in his right kidney without any evidence of contrast enhancement. The mass was separated from the kidney and appeared to have surrounded the kidney. There was no evidence of intrarenal mass on the CT scan. He had abdominal aortography and right renal angiography which revealed no evidence of hypervascular tumour. He underwent right radical nephrectomy and macroscopic examination of the specimen revealed that the tumour had originated from the perinephric fat and was separated from the kidney. The tumour had compressed the ureter causing hydronephrosis. The pathological examination of the specimen revealed features that were considered to be consistent with a diagnosis of angiomyolipoma (AML) of the perinephric space and this showed mature fat, blood vessels, and smooth muscle. His follow-up outcome data was not available.

Gupta et al. [4] in 2007 reported an 80-year-old woman who presented with abdominal pain. She had CT scan and MRI scan of abdomen and pelvis which showed a mass in her left perinephric space. She underwent left radical nephrectomy with excision of a 16 cm mass. Macroscopic examination of the specimen revealed a perinephric mass with cystic areas that contained dilated vascular spaces intermingled with necrotic tissue which had alternated with more solid, better preserved areas which contained spindled cells with elongated and hyperchromatic nuclei. Immunohistochemistry of the tumour showed positive staining for HMB-45 and focally for smooth muscle actin but negative staining was shown for chromogranin, synaptophysin, epithelial membrane antigen, vimentin, carcinoembryonic antigen, S100, desmin, CD45, CD20, and cytokeratin. The features of the pathological examination of the specimen were adjudged to be consistent with angiomyolipoma (AML) with atypical features in the retroperitoneum. At her 1-year follow-up she was found to have distant metastases in the bone and liver.

Gupta and Guleira [10] in 2011 reported a 42-year-old man who presented with abdominal pain. He had ultrasound scan and CT scan of the abdomen and pelvis which revealed a 220 cubic centimetres mass in his right adrenal space. He underwent renal sparing excision of the lesion and pathological examination finding of the tumour was consistent with angiomyolipoma (AML). His follow-up outcome data was not available.

**Figure 1:**
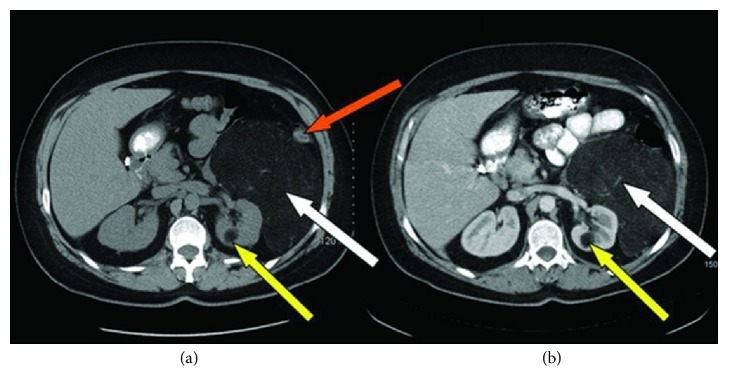
(a) Oral contrast and (b) IV and oral contrast: abdominal computerized tomography demonstrating an encapsulated fatty vascular mass (white arrows) lateral to the left kidney measuring 19.3 cm × 13.5 cm × 10.7 cm with prominent vascular dependence on the left renal vein and artery as well as a 2 cm posterior midpole homogeneous fatty density (yellow arrow). Left colon is laterally displaced (orange arrow). Reproduced from [18].

**Figure 2:**
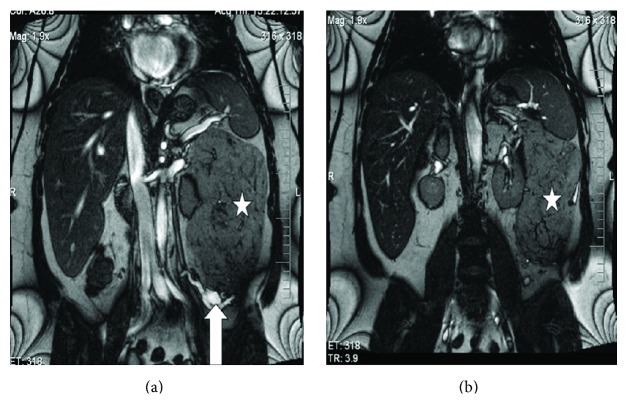
Abdominal magnetic resonance imaging demonstrating a large fatty encapsulated mass (white asterisk) measuring 19.3 cm × 13.5 cm × 10.7 cm with prominent vascularity (white arrows). The anatomic relationship between the mass and the left kidney can be well seen in Figure 2(b). Reproduced from [18].

**Figure 3:**
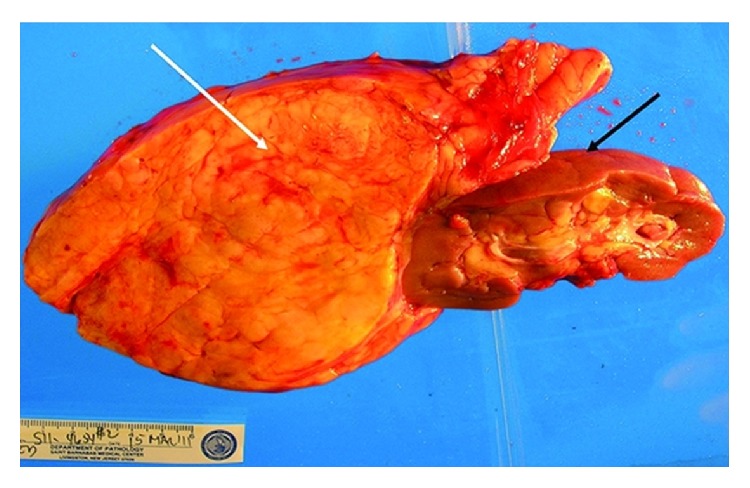
Gross image of the en bloc resected mass including the left kidney (black arrow), demonstrating a well-encapsulated fatty mass attached to the upper pole of the kidney (white arrow), with a smooth outer surface measuring 23 cm × 14 cm × 9 cm. Reproduced from [18].

**Figure 4:**
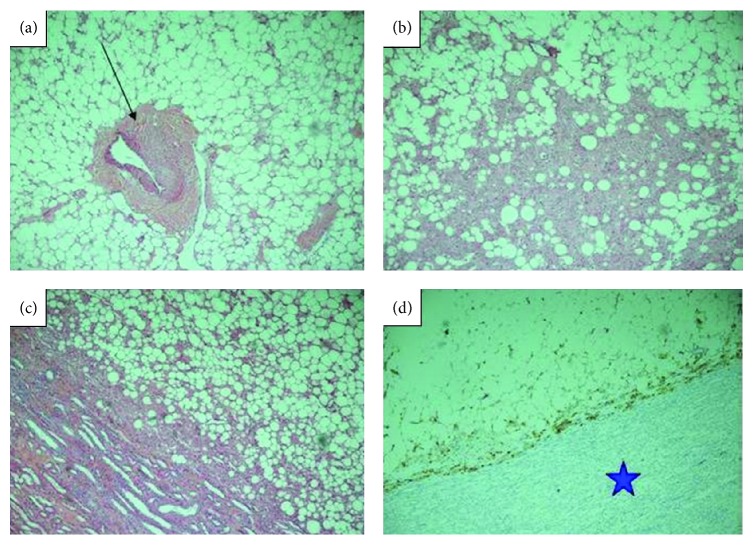
Extrarenal mass (haematoxylin and eosin). Photomicrograph of the mass demonstrate mature adipose tissue with a tortuous thick blood vessel (black arrow) ((a); ×20) and bundles of smooth muscles lacking elastic tissue lamina ((b); ×40), adipose tissue with small areas of smooth muscle with epithelioid features ((c); ×40). Focal staining with HMB45 antibody was positive (blue star) ((d); ×40), consistent with angiomyolipoma. Reproduced from [18].

Minja et al. [18] in 2012 reported the case of a 39-year-old woman who in 2011 had initially presented with dysfunctional uterine bleeding and who had transvaginal ultrasound scan which was normal. She developed protracted respiratory tract infection for which she had been on antibiotics and because of the unexpected duration of her symptoms she had CT scan of the thorax which revealed unremarkable findings in the chest. However, the lower Ct images of the chest did reveal a large retroperitoneal mass which had abutted the left kidney. She subsequently had contrast enhanced CT scan of the abdomen which showed an encapsulated mass that measured 19.3 cm × 13.5 cm × 10.7 cm with associated prominent vascularity which arose from the left renal vasculature. Additionally, there was evidence of a 2 cm homogeneous fatty renal lesion in the inferior midpole which was adjudged to be consistent with angiomyolipoma of the kidney (see Figures 1(a) and 1(b)). She also had MRI scan of abdomen and pelvis which showed a well-encapsulated fatty tumour that had displaced the left hemicolon laterally and this measured 19 cm × 14.4 cm × 13.8 cm. The mass was seen to have abutted tightly to the upper pole of the left kidney as well as a small 2 cm lesion was shown inferiorly which was considered to be most likely representing an angiomyolipoma of the kidney (see Figures 2(a) and 2(b)). Minja et al. [18] considered a number of differential diagnoses which included a retroperitoneal liposarcoma, leiomyosarcoma, lipoma, angiomyolipoma, adrenal adenocarcinoma, renal cell carcinoma, or leiomyoma with fatty change. She was asymptomatic with regard to the mass. Minja et al. [18] stated that the patient underwent en bloc resection of the mass with the left kidney via midline incision as well as total abdominal hysterectomy to treat her dysfunctional uterine bleeding contemporaneously. The kidney measured 11.5 cm × 4.5 cm × 3 cm and the mass which was situated near the upper pole of the kidney measured 23 cm × 14 cm × 9 cm (see Figure 3). Serial sections of the mass and kidney did reveal the lesion to be well and fully circumscribed and separate from the parenchyma of the kidney. Gross examination revealed the mass to be homogeneously yellow without any stigmata of necrosis or haemorrhage. Another well-circumscribed, intrarenal mass, which measured 2 cm × 1.8 cm × 1 cm, was also found within the lower midportion of the renal cortex. Macroscopic and microscopic examinations of the two lesions revealed similar features in that they had predominance of adipose tissue and smaller areas of smooth muscle with epithelioid features and characteristically abnormal vessels. The larger lesion was separate and distinct from the parenchyma of the kidney. Immunohistochemistry study of the larger lesion showed positive staining for HMB-45 which is characteristic for an angiomyolipoma (see Figure 4). Microscopic examinations of the uterus and cervix were normal. At her 16-month follow-up she was alive and asymptomatic. Minja et al. [18] stated that presence of perivascular epithelioid cells (PEC) tends often to be used to characterize angiomyolipomas in view of the fact that these cells exhibit immunoreactivity for muscle markers (epithelial membrane antigen, keratin, vimentin, desmin, and actin) and HMB-45 [68]. It has been stated that positive immunoreactivity for HM-45, a monoclonal antibody which has been raised against melanoma-associated antigen, is characteristic of angiomyolipomas (AMLs) and it can be used to differentiate angiomyolipomas (AMLs) from other similar appearing lesions, for example, liposarcomas, lipomas, leiomyosarcomas, or leiomyomas [11, 17].

Lee et al. [19] reported a 35-year-old woman who presented with symptoms of increased abdominal circumference and urinary frequency. She had a CT scan of abdomen which showed a 24 cm × 21 cm × 16 cm retroperitoneal fatty tumour which had displaced the right kidney to the left upper quadrant of the abdomen. She underwent laparotomy and wide excision of the tumour and preservation of the right kidney. Pathological examination of the excised tumour revealed features consistent with the diagnosis of angiomyolipoma (AML). At her 4-month follow-up, she was well with no evidence of tumour recurrence and her right kidney was functioning well.

Soni et al. [20] reported a 33-year-old woman who presented with left sided flank pain. She had CT scan of abdomen which showed a large angiomyolipoma that measured 9.3 cm × 6.1 cm × 7.7 cm located in the lower pole of the kidney. During her surgical operation the tumour was found to be located in a pararenal retroperitoneal position without involving the kidney. Surgical excision of the mass was undertaken with preservation of the kidney. Pathological examination of the excised tumour revealed predominantly adipose tissue with multiple thick-walled vascular channels that were lined by flattened endothelial cells. Perivascular epithelioid cells were proliferating and emanated from blood vessel wall and had extended into the surrounding adipose tissue. Immunohistochemical staining of the tumour showed strong positivity for HMB-45 and smooth muscle actin. The pathological examination findings were consistent with a diagnosis of pararenal angiomyolipoma. She was evaluated for evidence of tuberous sclerosis and this was negative. There was no long-term follow-up data on the patient.

Ivanova et al. [21] reported a 33-year-old woman who presented with dull pain in the right lumbar region. She had radiological imaging studies which showed a tumour mass in the right retroperitoneal space without any clear evidence of the kidney. During her surgical operation an ill-defined tumour mass was found to have encased the right kidney and hence an en bloc resection of the mass was undertaken. Macroscopic examination of the excised specimen showed an intact kidney which measured 10 cm × 6 cm and which was surrounded by multinodular, yellowish soft tumour which added about 6 cm to each side of the kidney. Microscopic examination of the specimen showed mature fat, an unevenly dispersed abnormal blood vessel of variable thickness, and spindle cell component which appeared to irradiate from vessel walls, which occasionally fused into solid areas with hyperchromatic nuclei. Neoplastic nests with similar appearance were observed within the superficial part of the renal cortex. Multiple small foci appeared to contain larger polygonal cells which had abundant clear cytoplasm which had epithelioid appearance. Few of the cells had exhibited increased nuclear atypia, and almost no evidence of mitotic figures was observed. A provisional diagnosis of liposarcoma was made based upon the high fat content, presence of spindle, and epithelioid cells which had mildly atypical nuclei and rare whirling pattern of spindle cell growth. Nevertheless, second opinion evaluation led to immunohistochemistry studies of the tumour which showed negative staining for cytokeratin AE1-AE3 and S100 protein but positive staining in scattered majority of the cells for HMB-45 and furthermore synchronous strong positive expression of smooth muscle actin was observed. A final diagnosis of angiomyolipoma (AML) was established. There was no follow-up data on the long-term outcome of the patient.

Welling et al. [22] reported a 38-year-old man who had melanoma of the right flank region. He had a staging 18F-fluoro-2-doxyglucose (FDG) PET/CT scan which showed a hypermetabolic extrarenal mass in the left retroperitoneal space which was reported to be concerning for metastatic melanoma. Nevertheless, pathological examination of the mass revealed features, that were adjudged to be consistent with angiomyolipoma (AML). Welling et al. [22] stated that angiomyolipomas (AMLs) tend to have a variety of radiological imaging appearances on multiple imaging modalities, including FDG PET, and can confound accurate diagnosis when the mass is in an extrarenal location. They also stated that their case had demonstrated the only known description of an extrarenal retroperitoneal angiomyolipoma (ERAML) and this would highlight the challenge of accurate diagnosis based upon FDG PET findings.

Fegan et al. [23] in 1997 reported a case of extrarenal retroperitoneal AML. The details of the case are not available but Fegan et al. [23] stated that their case was the 5th case of extrarenal retroperitoneal angiomyolipoma (ERAML) to be reported and in their case as well as in the other four previously reported cases, the correct diagnosis was made only after laparotomy, despite a number of prospective imaging studies. They suggested that careful exploration should in the future result in more renal sparing approaches to the management of extrarenal retroperitoneal angiomyolipoma (ERAML).

Lau et al. [24] in 2003 reported a patient who had primary retroperitoneal monotypic epithelioid angiomyolipoma which was composed exclusively of atypical epithelioid cells which had subsequently metastasized to the liver and the mediastinum. Lau et al. [24] stated that monotypic epithelioid angiomyolipoma has generally been considered to be a benign neoplasm even though rare cases of such lesions exhibiting malignant behaviour had been reported. They also stated that to their knowledge their case was the first report of metastatic disease which had occurred in an extrarenal retroperitoneal angiomyolipoma (ERAML).

Wen et al. [25] reported a 45-year-old woman with a history of tuberous sclerosis and left renal angiomyolipoma and had 15 years earlier undergone left radical nephrectomy and preoperative embolization of left kidney due to a huge AML which had compressed the descending colon to the right side and caused loss of appetite and constipation. Pathological examination of the specimen confirmed angiomyolipoma (AML) of left kidney without invasion of the renal vessel. She presented 15 years after her left nephrectomy with loss of appetite, vomiting, abdominal pain, and constipation. She was found on examination to have a palpable, huge, painless mass which had elastic to firm consistency and adenoma sebaceum skin lesions in the malar regions of her face. Her urinalysis and blood biochemistry tests were normal. She had CT scan which showed an adhesion ileus, a giant retroperitoneal tumour in the left side that had extended over the right retroperitoneum, and multifocal lipomatosis that had involved the posterior mediastinum, right kidney, and liver. The CT scan also revealed multiple pulmonary nodules in the right, middle, and the lower lobes of the lung and a focal right pneumothorax at the area of the pericardium. She underwent laparotomy during which a brown to yellowish tumour that had a fibrotic capsule and tortuous vessels was found in the entire left retroperitoneum. The tumour mass was excised and the right kidney was left intact. Pathological examination of the specimen did show that the tumour comprised of mature adipose tissue, large blood vessels which had thick muscular walls, and a number of foci that showed epithelioid cells surrounding the vessels. All the pathological features were adjudged to be consistent with the diagnosis of angiomyolipoma (AML). She did not have any gastrointestinal symptoms for one year.

Wang et al. [26] reported a 48-year-old woman who presented with abdominal pain and weight loss. On examination she was found to have a palpable mass in the left side of her abdomen. She was referred from her initial hospital after she had had a CT scan which was provisionally reported as showing features suggestive of a retroperitoneal liposarcoma. She had another CT scan of abdomen in the second hospital on her admission which revealed a huge left sided retroperitoneal fatty tumour which had encompassed the left kidney. The CT scans also showed that the tumour had extended caudally into the pelvis, anterior to the uterus, and many intrarenal fatty nodules that were clearly separated from huge tumour and all these radiological findings were adjudged to be consistent with the diagnosis of retroperitoneal angiomyolipoma (RAML). Four days after her admission she developed sudden onset of severe abdominal pain which was ensued by her development of shock for which she was resuscitated and for which she underwent an emergency laparotomy which revealed that she had developed spontaneous haemorrhage of her large retroperitoneal angiomyolipoma (RAML). Excision of the mass as well as left nephrectomy was undertaken. The pathological examination of the specimen did confirm the radiological diagnosis of retroperitoneal angiomyolipoma. Microscopic examination of the specimen also showed scattered small angiomyolipomas of the left kidney. The case was reported without any long-term follow-up information which would indicate that the case was published not long after the patient was discharged.

Daniel et al. [27] reported a 34-year-old woman who underwent excision of a large left retroperitoneal mass which had extended between the diaphragm and the bifurcation of the iliac vessels. They stated that the pathological features of the lesion were consistent with the diagnosis of retroperitoneal angiomyolipoma (RAML) and their reported case was the 19th case of extrarenal retroperitoneal angiomyolipoma (ERAML) to be reported.

Vîlcea et al. [28] reported a 65-year-old man who presented with an irreducible tumour in the right inguinofemoral region which he had had for four years with no subjective symptoms. A provisional preoperative diagnosis of irreducible right inguinal hernia was made. During the surgical procedure upon the inguinal canal a lipomatous mass was found which was in continuity with the scrotum and which was continuous through the deep inguinal canal (internal ring) with a retroperitoneal tumour. Two ureters in the right inguinal canal as well as absence of a peritoneal sac were observed. The lipomatous mass was excised en bloc with the right testis; nevertheless, the section of the tumour was limited to the deep inguinal (internal) ring. Both ureters were reduced into the retroperitoneal space. Postoperatively, he had a CT scan of abdomen and pelvis which showed a 15 cm × 25 cm inhomogeneous retroperitoneal tumour, the location of which had extended from the lower pole of the kidney to the inguinal arch. The CT scan also showed that the tumour contained fat tissue densities, areas of haemorrhage in the tumour, calcifications, and sclerosis; the tumour had pushed the right kidney superiorly and laterally and included two ureteric ducts. Furthermore, the CT scan did show thickening of the pararenal fascia, cleavage plane with the inferior vena cava, and abdominal wall, and it showed right sided hydronephrosis. He had an intravenous urography which showed grade 2 right sided hydronephrosis and a single right sided ureter which was amputated at the level of the iliac crest. He underwent another laparotomy three weeks later which revealed the following: a 15 cm × 25 cm lipomatous tumour extending caudally from the lower pole of the right kidney; the tumour had encased two ureteric ducts (one of the ureters had originated from the right renal pelvis and the second ureter had developed from the upper pole of the right kidney without any relationship to the renal pelvis). At laparotomy the tumour was found to have smooth, elastic areas, which were adjudged to be specific to a lipoma, but the distal part of the tumour was found to be hardened and at that level (the distal part) the surgeon found dissection of the ureter impossible. The tumour was excised en bloc with both ureters and the kidney. Macroscopic examination of the excised specimen showed a 15 cm × 25 cm mass which had lipomatous appearance and areas of haemorrhage that had alternated with areas of necrosis as well as hardened areas with sclerosis and calcifications; the two right ureters were stuck tightly in the fibrosclerosis process. Pathological examination of the specimen showed a mature adipose tissue admixed with smooth muscle tissue proliferation, occasional giant cells that surrounded medium calibre blood vessels. The microscopic features of the tumour were adjudged to be conclusive with a diagnosis of angiomyolipoma (AML). At his 7-year follow-up, the patient was alive and well with no evidence of recurrence of his disease.

Mansi et al. [29] in 2002 reported a case of a large extrarenal angiomyolipoma which had mimicked a large locally advanced renal parenchymal tumour. They stated that the diagnosis by means of histopathology examination of the specimen after radical nephrectomy had been performed and that even though angiomyolipoma (AML) is rare, angiomyolipoma (AML) of the perinephric fat may present in various ways and should be considered in the differential diagnosis of large renal tumours especially in view of the possibility of kidney sparing management.

Ahmad et al. [30] reported a 44-year-old woman who presented with right sided back pain. She had MRI scan and CT scan which showed a 10 cm retroperitoneal mass lying posterior to the right kidney and pushing the kidney anteriorly, and the mass was not connected to the kidney, adrenal gland, vascular, or other structures. She had CT scan guided biopsy but the histological features of the specimen were nondiagnostic. She underwent excision of the mass which was well-encapsulated, firm, and ovoid and measured 14 cm × 10 cm × 6 cm with fibrofatty tissue. Macroscopic examination of the specimen revealed a lobulated grey homogeneous tumour with a cystic area in a peripheral location. Microscopic examination of the specimen showed myoid spindle proliferation which was arranged in irregular sheets and nests that had been separated by prominent stromal hyalinization. The tumour cells exhibited vacuolated to eosinophilic cytoplasm with pink globules. In some areas there was evidence of dense hypercellularity and fascicular arrangement. Radial and concentric arrangement of the tumour cells was seen around thick-walled medium sized malformed blood vessels. There was evidence of mild nuclear pleomorphism and mitosis was rare. There was no tumour necrosis, vascular invasion, or infiltrative growth pattern. Immunohistochemistry revealed diffuse positive staining for desmin and smooth muscle actin, and scattered positivity for HMB-45 as well as S-100 protein. CD34 highlighted the vascular endothelial lining but the tumour cells were negatively stained. Immunohistochemistry of the tumour was negative for Melan-A, CD99, Cd10, inhibin, calretinin, and pancytokeratin (CK AE1/AE3). The pathological features of the tumour were diagnostic of angiomyolipoma (AML).

Molina et al. [31] reported a 28-year-old woman in her first pregnancy who presented with loin pain during the 17th week of her pregnancy which eventually resulted in spontaneous abortion and retained placenta. She was managed for the complications of her spontaneous abortion and her radiological imaging studies including ultrasound scan and CT scan showed a large mass in the left side of her retroperitoneum abutting the Gerota fascia of the left kidney displacing the left kidney. The mass was located between the splenic flexure and the iliac region. She underwent excision of the mass and pathological examination of the excised specimen showed features consistent with extrarenal retroperitoneal angiomyolipoma.

Medina et al. [32] reported a 25-year-old patient who presented with abdominal distension and whose ultrasound scan of abdomen had shown a large solid hypoechogenic mass which had occupied the whole pelvis and extended to the umbilical region displacing the adjacent organs. A CT scan which was subsequently done confirmed presence of the mass. An imaging guided per-cutaneous biopsy of the mass was undertaken and pathological examination of the specimen confirmed extrarenal retroperitoneal angiomyolipoma (ERAML).

Peces et al. [69] reported a 40-year-old man with a history of sporadic tuberous sclerosis and with a history of spontaneous bleeding from his left kidney angiomyolipoma. He received a low-dose mTor inhibitor and rapamycin for 12 months and this was noted to be associated with a reduction in the volume of his bilateral angiomyolipomas (AMLs) and it was noted to have resulted in stabilization as well as improvement of his renal function. Furthermore, there was additionally a reduction of his facial angiofibromas, improvement in the control of his blood pressure, and absence of angiomyolipoma (AML) bleeding over the 12-month period. His tuberous sclerosis brain lesion images did remain stable, and there was no significant rapamycin associated side-effects. Peces et al. [69] stated that to the best of their knowledge, their reported case was the first reported case of reduction in the volume of angiomyolipoma (AML) together with preservation of renal function in a patient with tuberous sclerosis who had received low-dose rapamycin. They also iterated that these data would suggest that it could be the result of the antiangiogenic, antifibrotic, and antiproliferative effects of rapamycin.

It is known that in tuberous sclerosis a number of tumours develop in various parts of the patient's body. A case of retroperitoneal angiomyolipoma has been reported in a patient 15 years after the patient had undergone radical nephrectomy. It could be argued that the new reported angiomyolipoma was a de novo benign angiomyolipoma due to the fact that the patient had tuberous sclerosis. On the contrary some people could argue that perhaps the newly reported angiomyolipoma could have been a metastatic angiomyolipoma that developed very late. Furthermore, two cases of distant metastases have been reported in two patients following radical resection of their retroperitoneal angiomyolipomas. Some people would argue that if angiomyolipomas are benign tumours then the subsequent development of angiomyolipomas in the liver, bone, and mediastinum could be considered as the subsequent development of de novo benign angiomyolipomas in other sites. However, it could be argued that the development of distant metastases occurring in a nontuberous sclerosis patient is the development of true metastases rather than de novo primary tumours. If that is the case, it would further be argued that there is the need for academic pathologists and oncologists globally to have a consensus opinion meeting discussion on angiomyolipomas to decide whether angiomyolipomas should still be regarded as benign tumours or they should be regarded as slow-growing malignant tumours. There is also the need to discuss further if in event of reclassification of angiomyolipomas as potentially slow-growing malignant tumours should patients undergoing selective embolization or surgical resection of angiomyolipomas be given adjuvant rapamycin.

There is no consensus opinion on what angiomyolipomas occurring in the retroperitoneum. Some authors have referred to the lesions as retroperitoneal angiomyolipomas but other authors have referred to the lesions as extrarenal retroperitoneal angiomyolipomas. Some people would argue that the kidney lies in the retroperitoneum and thus angiomyolipomas of the kidney are also retroperitoneal angiomyolipomas and that by using the terminology extrarenal retroperitoneal angiomyolipoma every one would understand that the angiomyolipoma does not involve the kidney. Those who use the terminology retroperitoneal angiomyolipoma use the terminology knowing that angiomyolipomas of the kidney are strictly confined to the kidney and should not be regarded as retroperitoneal angiomyolipomas. With regard to the two different terminologies used in the literature, the author has observed from a review of the literature that all angiomyolipomas of the kidney have always been referred to as angiomyolipomas of the kidney or renal angiomyolipomas and these lesions had not been regarded as retroperitoneal angiomyolipomas even though the kidney could be said to be located in the retroperitoneum.

## 4. Conclusions and Recommendations

Extrarenal retroperitoneal angiomyolipoma (ERAML) is a rare tumour which could be confused with other tumours; ERAML was previously regarded as a hamartoma but is now classified as a benign tumour. Considering the fact that there are reports of the occurrence of distant metastases following complete excision of ERAML it would be recommended that academic pathologists and oncologists globally should convene a consensus opinion meeting to discuss the pathology and biological behaviour of the lesion in order to classify ERAML as a malignant neoplasm which tends usually to exhibit benign biological behaviour but has the potential to metastasize. Pathologists globally should also review the pathological features of ERAML that would indicate the possibility of malignant biological behaviour of the lesion. In view of the fact that there is evidence to show that mTor inhibitor rapamycin has been reported to be associated with reduction of the volume of angiomyolipoma (AML), perhaps it would be a good idea to give all patients who have undergone embolization of resection of angiomyolipoma (AML) as adjuvant treatment. Perhaps patients with extrarenal retroperitoneal angiomyolipoma (ERAML) who are not fit to undergo surgical procedures should be considered for rapamycin treatment.

## Figures and Tables

**Table 1:** A table of reported cases of extrarenal retroperitoneal angiomyolipoma.

Case	Reference	Presentation	Age (years)/ sex	Size	Imaging	Location	Treatment	Outcome/follow-up
1	Friis and Hjortrup 1982 [5]	Pain and weight gain	22/ female	11 kilograms	Intravenous urography	Peripancreatic space	Radical nephrectomy	Asymptomatic/36 months

2	Randazzo et al. 1987 [6]	Pain and bleeding	64/ female	6 cubic cms	IVU and CT scan	Right perinephric space	Renal sparing surgery	Asymptomatic/2 months

3	Ditonno et al. 1992 [7]	Pain and bleeding	37/ male	5 cm	IVU and CT scan and angiography	Right perinephric space	Radical nephrectomy	Details not available

4	Peh et al. 1994 [8]	Abdominal mass and weight loss	32/ female	3.7 kilograms (7980 cubic cms)	Ultrasound scan and CT scan	Left perinephric space	Radical nephrectomy	Asymptomatic/8 months

5	Angulo et al. 1994 [9]	Abdominal pain and flank pain	53/ female	336 cubic cms	Ultrasound scan, CT scan, and angiography	Left perinephric space	Radical nephrectomy	Details not available

6	Gupta and Guleria 2011 [10]	Abdominal pain	42/ Male	220 cubic cms.	Ultrasound scan and CT scan	Right adrenal space	Renal sparing resection	Details not available

7	Liwnicz et al. 1994 [11]	Abdominal pain	39/ female	1.1 kilograms (216 cubic cm)	CT scan	Right perinephric space	Radical nephrectomy	Asymptomatic/18 months

8	Law et al. 1994 [12]	Incidental finding	59/ female	22.5 cubic cm	CT scan and MRI scan	Left perinephric space	Radical nephrectomy	Details not available

9	Law et al. 1994 [12]	Pain	56/ female	11 cm	IVU, CT Scan, ultrasound and fine needle aspiration	Left perinephric space	Radical nephrectomy	Asymptomatic/8 months

10	Mogi et al. 1998 [13]	Abdominal fullness and pain	41/ female	648 cubic cm	CT scan and MRI scan	Right perinephric space and perihepatic space	Renal sparing resection of mass	Details not available

11	Murphy et al. 2000 [14]	Abdominal pain and bleeding	51/ female	Details not described	CT scan and angiography	Left perinephric space	Selective angiography embolization	Asymptomatic/12 months

12	Tsutsumi et al. 2001 [15]	Abdominal pain and fatigue (lethargy)	60/ female	3.5 kilograms (4840 cubic cm)	CT scan and angiography	Right perinephric space	Radical nephrectomy	Asymptomatic/60 months

13	Tseng et al. 2004 [16]	Fullness of abdomen	35/ female	2.8 kilograms (3726 cubic cm)	Ultrasound scan, CT scan and angiography	Right perinephric space	Renal sparing resection	Details not available

14	Obara et al. 2005 [17]	Visible haematuria	31/ male	Details not documented	CT scan and angiography	Right perinephric space	Radical nephrectomy	Details not available

15	Gupta et al. 2007 [4]	Abdominal pain	80/ female	16 cm	CT scan and MRI scan	Left perinephric space	Radical nephrectomy	Distant metastases in bone and liver/12 months

16	Minja et al. 2012 [18]	Asymptomatic	39/ female	1.7 kilograms (2898 cubic cm)	Ultrasound scan/CT scan	Left perinephric space	Radical nephrectomy	Asymptomatic/16 months

17	Lee et al. 2003 [19]	Increasing abdominal circumference and urinary frequency	35/ female	24 cm × 21 cm × 16 cm	CT scan	Right retroperitoneal region displacing right kidney to left	Wide excision with preservation of kidney (kidney sparing resection of mass)	Asymptomatic/4 months

18	Soni et al. 2013 [20]	Left sided abdominal pain	33/ female	9.3 cm × 6.1 cm × 7.7 cm	CT scan	Left pararenal retroperitoneal space	Kidney preservation excision of mass	Follow-up information was not available

19	Ivanova et al. 2014 [21]	Right sided lumbar ache	33/ female	10 cm × 6 cm	Radiological imaging type not available	Right retroperitoneal space	Kidney preserving resection	Follow-up information was not available

20	Welling et al. 2012 [22]	Incidental finding of staging FDG PET/CT scan for melanoma of right flank	38/ male	Hypermetabolic extrarenal mass size not available	FDG PET/CT scan done as staging CT for melanoma	Left retroperitoneal space	Details of surgery not available	Follow-up information not available

21	Fegan et al. 1997 [23]	Details not available	Details not available	Details not available	Details not available	Extrarenal retroperitoneal details not available	Details not available	Details not available

22	Lau et al. 2003 [24]	Details not available	Details not available	Details not available	Details not available	Extrarenal retroperitoneal details not available	Details not available	Metastases reported in the abstract to the liver and mediastinum (the questions needed to be asked: are the lesions true metastases? Or are they de novo primaries in the liver and mediastinum? If they are metastases then should angiomyolipoma not be classified as malignant tumours?)

23	Wen et al. 2014 [25]	History of tuberous sclerosis and left renal AML had preoperative embolization plus left nephrectomy 15 years earlier. Presented with loss of appetite, vomiting, abdominal pain, constipation, and palpable mass in abdomen	45/ female	Large right extrarenal retroperitoneal mass and lesions in other sites	CT scan	Right retroperitoneal AML and lesions elsewhere	Radical excision of mass sparing the kidney	Asymptomatic at 1-year follow-up (in view of the fact that patients with tuberous sclerosis have a tendency to develop lesions in multiple sites, it would be argued that the ERAML that developed 15 years later was a de novo lesion rather than metastatic lesion. On the contrary other people could argue that perhaps the lesion was a late metastatic lesion).

24	Wang et al. 1997 [26]	Abdominal pain and weight loss plus spontaneous bleeding in the tumour	48/ female	Size not available	CT scan	Left retroperitoneal space	Left nephrectomy together with excision of the mass	Follow-up information not available

25	Daniel et al. 2010 [27]	Details not available	34/ female	Large	No available information on imaging	Left retroperitoneal space		Follow-up details not available

26	Vîlcea et al. 2015 [28]	Irreducible right inguinofemoral mass considered to be irreducible hernia	65/ male	Large; 15 cm × 25 cm	CT scan and intravenous urography	Large right retroperitoneal mass down to inguinal femoral canal and pushing away nearby organs	Wide excision	Asymptomatic/7 years

27	Mansi et al. 2002 [29]	Details not available	Details not available	Details not available but large	Details not available	Retroperitoneal but details not available		Details not available

28	Ahmad et al. 2006 [30]	Right back pain	44/ female	14 cm × 10 cm × 6 cm	MRI scan; CT guided biopsy of mass	Right retroperitoneal	Excision of the mass	Follow-up details not available

29	Molina et al. 2001 [31]	Spontaneous abortion in first pregnancy associated with complications; diagnosis was incidentally made during imaging investigations	28/ female	Size not available	Ultrasound scan and CT scan	Left retroperitoneum	Excision of mass	Follow-up information not available

30	Medina et al. 2002 [32]	Abdominal distension	25/ sex of patient not available	Large extending from pelvis to umbilical region	Ultrasound scan and CT scan and radiological imaging biopsy	Retroperitoneal space extending from pelvis to umbilical region	Details of treatment not available	Follow-up details not available
